# Clinical significance of high-dose chemotherapy with autologous stem cell transplantation in the era of novel agents in patients older than 65 years with multiple myeloma

**DOI:** 10.1007/s00277-023-05177-7

**Published:** 2023-03-23

**Authors:** Shuku Sato, Shun Tsunoda, Teiko Kawahigashi, Wataru Kamata, Yotaro Tamai

**Affiliations:** Division of Hematology, Shonan Kamakura General Hospital, 1370-1 Okamoto, Kamakura, Kanagawa 247-0072 Japan

**Keywords:** Autologous stem cell transplantation, Multiple myeloma, Older patients, Complete response, Progression-free survival

## Abstract

High-dose chemotherapy followed by autologous stem cell transplantation (ASCT) is the standard treatment for symptomatic multiple myeloma (MM) in patients under 65 years of age. However, the performing of ASCT in older patients > 65 years without comorbidities or complications is controversial. Introduction of novel drugs, such as daratumumab, has improved the long-term survival of patients with MM who are ineligible for ASCT. This retrospective study aimed to evaluate the clinical significance of ASCT in older patients, even in the era of novel drugs. A total of 55 patients aged 65–74 years (15 ASCT recipients and 40 ASCT-ineligible patients) newly diagnosed with MM between March 2013 and October 2021 at our institution were analyzed in this study. There were no significant differences in the 3-year overall survival (84.6% vs. 90.6%, *p* = 0.72) and progression-free survival (PFS) (61.2% vs. 75.1%, *p* = 0.40) between ASCT recipients and ASCT-ineligible patients. There was also no significant difference in complete response (CR) with minimal residual disease (MRD)-negative rate between the two groups (27% vs. 33%, *p* = 1.0). Multivariate analysis showed that CR was an independent predictor of PFS (hazard ratio [HR], 0.26; 95% confidence interval, 0.08–0.76; *p* = 0.01). In this retrospective study, despite patients who were determined to be intolerant to ASCT, the non-ASCT group was non-inferior to the ASCT group in PFS and overall response rate. The results of this study confirm that the significance of ASCT is diminishing in patients 65 years of age and older because newer agents can achieve good responses without ASCT.

## Introduction

In the IFM 2009 trial, combination therapy with bortezomib, lenalidomide, and dexamethasone (VRd) plus high-dose chemotherapy followed by autologous stem cell transplant (ASCT) was reported to be significantly superior to VRd alone in terms of overall response rates (ORRs) and progression-free survival (PFS) [1]. Furthermore, in the phase III DETERMINATION trial, addition of ASCT to VRd induction therapy with ongoing lenalidomide maintenance showed prolonged PFS in the VRd plus ASCT group compared to that in the VRd alone group [2]. More than 30 years after its introduction, high-dose chemotherapy followed by ASCT remains the standard treatment for young patients under the age of 65 with newly diagnosed multiple myeloma (MM) according to the results of large randomized clinical trials [1, 3]. In addition, numerous reports have shown the efficacy and safety of ASCT in older MM patients over 65 years of age [1–9].

In contrast, daratumumab, lenalidomide, and dexamethasone (DRd) therapy have been recommended for newly diagnosed MM patients who are not eligible for transplantation since 2019 in Japan. In the MAIA trial, the median PFS of DRd therapy was not reached at 60 months, indirectly comparable to the transplant group in the IFM 2009 trial mentioned above [10].

Patients between the ages of 65 and 74 are the most common age group diagnosed with MM, accounting for 33% of all MM patients [11], and ASCT for newly diagnosed MM patients in this age group is currently controversial. This study aimed to evaluate the clinical significance of ASCT in newly diagnosed MM patients over 65 years of age, even in the era of novel agents, including anti-CD38 monoclonal antibody (mAb).

## Patients and methods

In this retrospective study, the medical records of 55 patients newly diagnosed with MM at Shonan Kamakura General Hospital between March 2013 and October 2021 were analyzed. This study was approved by the Research Ethics Committee of Shonan Kamakura General Hospital (ethics committee approval number: TGE02003-024). All the patients consented to the use of their medical records. This study was performed in accordance with the institutional guidelines and principles of the Declaration of Helsinki. We evaluated and compared the ORRs, overall survival (OS), and PFS at 3 years in the ASCT and non-ASCT (did not undergo ASCT) groups. The minimal residual disease (MRD)-negative and transplant-related mortality (TRM) rates were also evaluated.

In the ASCT group, seven patients received induction therapy with bortezomib and dexamethasone (Bd) and eight patients received induction therapy with VRd. Melphalan (140 mg/m^2^) was the conditioning regimen administered to all patients prior to ASCT. By contrast, in the non-ASCT group, 7 patients received daratumumab-based regimen, 17 patients received Bd, 11 patients received VRd, and 5 patients received lenalidomide and dexamethasone (Rd) as induction therapy. The rationale for using the doublet regimen was patient preference in the case of Rd therapy, while in the case of Bd therapy, lenalidomide could not be administered from the beginning owing to renal dysfunction. All patients provided informed consent regarding ASCT; we did not actively recommend ASCT to patients over 70 years of age, to those with performance status (PS) 2 or higher, or to those who had complications of infection during induction therapy owing to potential adverse events.

### Statistical analyses

OS was defined as the time from the start of the initial therapy to death from any cause. Patients who had not relapsed, progressed, or died were censored on the date of the last follow-up. The Kaplan–Meier method and a log-rank test were used to estimate and compare 3-year OS and PFS. All tests were two-sided, 95% confidence intervals (CI) were calculated, and *p*-values < 0.05 were considered statistically significant. The *χ*^2^ test or Fisher’s exact test was used to assess the differences in patient characteristics, including sex, renal dysfunction, international staging system, response, and recurrence in the ASCT or non-ASCT subgroups. The Cox proportional hazards model was used for the univariate and multivariate regression analyses. Statistical significance was set at* p* < 0.05. All analyses were performed using EZR (Saitama Medical Center, Jichi Medical University; http://www.jichi.ac.jp/saitama-sct/SaitamaHP.files.statemedEN.html; Kanda, 2012), which is a graphical user interface for R (The R Foundation for Statistical Computing, version 2.13.0), and a modified version of R commander designed to add statistical functions was used [12].

## Results

We analyzed the data of 55 patients (15 ASCT recipients and 40 ASCT-ineligible patients). The median age of the patients was 69.4 years (range 65–74), and the median observation period was 38.2 (2.1–109) months. The reasons why 40 patients did not receive ASCT were as follows: 30 patients declined transplantation, nine were excluded by the attending physician (two had comorbidities, three had poor performance status (PS), and four had infections during induction therapy), and one was a poor mobilizer. The clinical and laboratory features of MM patients with or without ASCT prior to treatment are summarized in Table 1. Patients in the ASCT group were younger; the PS and MM subtypes did not differ between the ASCT and non-ASCT groups. The ISS, R-ISS, LDH at initial presentation, myeloma-related events, and high-risk cytogenetic abnormalities (CA) were also similar between the two groups.

**Table 1:** Patient baseline disease characteristics

	ASCT *N* = 15	W/o ASCT *N* = 40	*p* value
Median age—yr (range)	66.7 (65–70)	70.7 (65–74)	< 0.001
Sex—no. (%)			
Male	6 (40.0)	24 (60.0)	0.231
Female	9 (60.0)	16 (40.0)	
ECOG performance status—no (%)			
0	12 (80.0)	22 (55.0)	0.31
1	1 (6.7)	7 (17.5)	
≥ 2	2 (13.3)	11 (27.5)	
Myeloma subtype—no (%)			
IgG	11 (73.3)	28 (70.0)	1
IgA	2 (13.3)	4 (10.0)	
BJP	2 (13.3)	7 (17.5)	
IgM	0 (0.0)	1 (2.5)	
International Staging System (ISS) stage—no. (%)			
I	4 (26.7)	9 (22.5)	0.682
II	5 (33.3)	19 (47.5)	
III	6 (40.0)	12 (30.0)	
Revised-ISS stage—no. (%)			
I	4 (26.7)	9 (22.5)	0.717
II	9 (60.0)	21 (52.5)	
III	2 (13.3)	10 (25.0)	
Lactate dehydrogenase ≥ 230 U/l—no. (%)	2 (13.3)	5 (13.5)	1
Creatinine clearance < 30 ml/min—no. (%)	2 (13.3)	12 (30.0)	0.304
History of bone lesions—no. (%)	10 (66.7)	27 (67.5)	1
Hemoglobin < 10 mg/dl—no. (%)	13 (86.7)	29 (72.5)	0.477
Hypercalcemia—no. (%)	2 (13.3)	6 (15.0)	1
Free light chain clonality > 100—no. (%)	8 (53.3)	19 (47.5)	0.768
High-risk cytogenetic abnormalities	4 (26.7)	14 (35.0)	0.749
t(4;14)	2 (13.3)	7 (17.5)	1
TP53	0 (0.0)	7 (17.5)	0.171
t(14;16)	2 (13.3)	2 (5.0)	0.298
Induction regimen			
Bd	8 (53.3)	17 (42.5)	0.236
VRd	7 (46.7)	11 (27.5)	
DRd or DVMP	0	7 (17.5)	
Rd	0	5 (12.5)	
Consolidation regimen or 2nd line regimen			
VRd	2 (13.3)	0	0.03
DRd, DPd, or DBd	1 (6.7)	16 (40.0)	
ELd or EPd	1 (6.7)	4 (10.0)	
Rd or lenalidomide alone	5 (33.3)	10 (25.0)	
IRd or Ixazomib alone	3 (20.0)	4 (10.0)	
KRd or weekly Kd	2 (13.3)	1 (2.5)	
Other	1 (6.7)	2 (5.0)	

*Bd* bortezomib dexamethasone, *VRd* bortezomib lenalidomide dexamethasone, *DRd* daratumumab lenalidomide dexamethasone, *DVMP* daratumumab bortezomib melphalan prednisolone, *Rd* lenalidomide dexamethasone, *DPd* daratumumab pomalidomide dexamethasone, *DBd* daratumumab bortezomib dexamethasone, *ELd* elotuzumab lenalidomide dexamethasone, *EPd* elutuzumab pomalidomide dexamethasone, *IRd* ixazomib lenalidomide dexamethasone, *KRd* carfilzomib lenalidomide dexamethasone, *Kd* calfilzomib dexamethasone

The complete response (CR) rate before ASCT was 13% and after ASCT 33%, but 60% of the patients in the ASCT group achieved CR with consolidation therapy with novel drugs after transplantation. The median time from diagnosis to transplant was 5.1 months (range: 4.2–8.9). Retrospectively, the median number of CD34^+^ cells was 2.5 × 10^6^/kg (range; 2.0–6.0). The median day of neutrophil engraftment was day 12 (range, 10–16), and the infection rate for ASCT was 73%. Day + 100 ASCT TRM was 0%, and ASCT could be safely performed in our hospital for older MM patients > 65 years. In contrast, in the non-ASCT group, the CR rate was 58%, similar to that in the ASCT group. Furthermore, when comparing the ASCT (*n* = 15), non-ASCT with daratumumab (that received daratumumab early for up to two lines; *n* = 17), and non-ASCT without daratumumab (*n* = 23) groups, the non-ASCT with daratumumab group had a significantly better CR rate (CR rates: ASCT group: 60%, non-ASCT with daratumumab group: 82.4%, non-ASCT without daratumumab group: 39.1%).

**Fig. 1:**
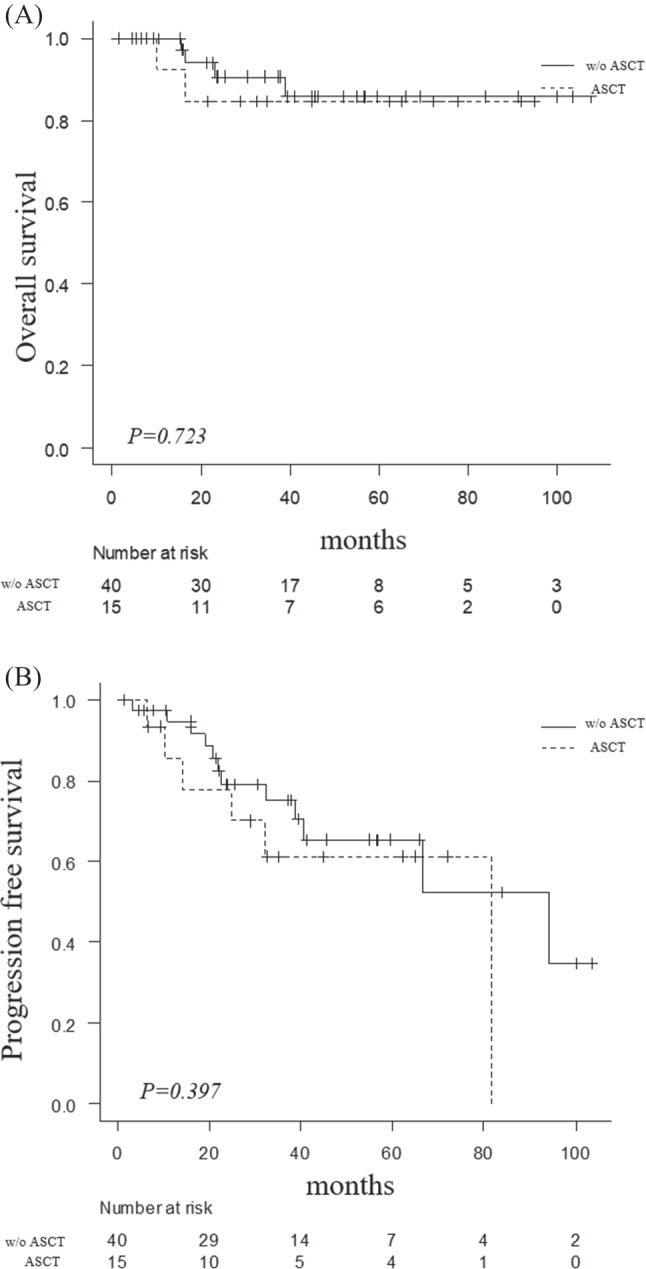
Outcomes of patients with MM who underwent ASCT or without (w/o) ASCT. Overall survival (**A**), progression-free survival (**B**)

The ORRs in ASCT groups and all non-ASCT group were similar (*p* = 1.00) (Table 2). Compared with the ASCT and non-ASCT groups, 3-year OS and PFS were similar (OS: 84.6% vs. 90.6%; *p* = 0.72, PFS: 61.2% vs. 75.1%; *p* = 0.40) (Fig. 1a, b). MRD was measured at the time of best response. Similar to the CR rate, the CR MRD-negative conversion rate was similar in both groups (27% vs. 33%, *p* = 1.00). However, when the non-ASCT group was examined more closely, the MRD-negative rate was 52.9% in the non-ASCT with daratumumab group, and 4.3% in the non-ASCT without daratumumab group; thus, the non-ASCT with daratumumab group had a significantly better MRD-negative rate than did the ASCT group (*p* = 0.002). In the non-ASCT group, MRD could not be fully examined because of the numerous cases in which MRD was not measured. According to the multivariate analysis (Table 3), CR was an independent predictor of PFS (hazard ratio [HR], 0.26; 95% CI, 0.08–0.76; *p* = 0.01), but ASCT was not an important factor in multivariate analysis.

**Table 2:** Best response and minimal residual disease status

	ASCT *n* = 15 *n* (%)		W/o ASCT *n* = 40 *n* (%)	
	After ASCT response	Best response	Best response	*p* value
Overall response	15 (100)	15 (100)	39 (97.5)	
Complete response	4 (26.6)	9(60)	23 (57.5)	1
Very good partial response	8 (53.3)	4 (26.7)	13 (32.5)	
Partial response	3 (20)	2 (13.3)	3 (7.5)	
Stable disease	0	0	1 ( 2.5)	
Negative status for MRD	2 (15.3)	4 (26.7)	10 (33)	1

*MRD* minimal residual disease

**Table 3:** Univariate analysis and multivariate analysis of prognostic factor for PFS

	Univariate analysis		Multivariate analysis	
	Hazard ratio	*p* value	Hazard ratio	*p* value
Age > 70	1.23 (0.48–3.11)	0.65		
Renal Cre > 2	2.85 (1.10–7.42)	0.03	0.84 (0.23–3.02)	0.79
Alb < 3.5	1.71 (0.64–4.62)	0.29		
LDH (> normal)	3.37 (0.92–12.3)	0.06	3.69 (0.85–16.0)	0.08
β2MG > 3.5	4.20 (1.21–14.7)	0.02	2.98 (0.62–14.2)	0.16
ASCT	1.53 (0.56–4.15)	0.40		
ISS = 3	4.80 (1.88–12.3)	0.001	1.79 (0.52–6.06)	0.35
R-ISS = 3	1.94 (0.91–4.14)	0.08		
CR MRD negative	0.56 (0.15–19.9)	0.37		
CR	0.36 (0.13–0.97)	0.04	0.26 (0.08–0.76)	0.01
No high-risk CA	3.28 (1.28–8.39)	0.01	2.18 (0.66–7.29)	0.20
Anti-CD38 mAb ≤ 2line	0.93 (0.30–2.96)	0.91		

*CR* complete response, *MRD* minimal residual disease, *CA* chromosome abnormalities

## Discussion

In this study, the ORR, CR rate, 3-year OS/PFS, and MRD-negative rates were similar between the ASCT and non-ASCT groups in patients aged 65–75 years. ASCT was not found to be significantly better in terms of response rates or PFS; therefore, ASCT cannot be implied to be significantly superior to non-ASCT. Notably, in this study, patients who received early line anti-CD38 mAb treatment achieved higher CR rates than those who did not receive ASCT, whereas patients who did not receive anti-CD38 mAb treatment did not have profound responses. In terms of patient background, patients who could undergo ASCT were in generally good condition to tolerate ASCT, while those in the non-ASCT group were in worse condition due to comorbidities, infections, and poor PS because many of them should have avoided transplantation. In addition, among the high-risk CA cases, TP53-positive cases were observed only in the non-ASCT group. Nevertheless, the ASCT group was not superior to the non-ASCT group in ORR and OS/PFS.

Multivariate analysis showed that ASCT did not contribute to prolonged PFS, and only CR achievement contributed to PFS. CR was achieved in 60% of the cases in both groups. In all other patients, with or without ASCT, achieving CR appears to be the most important for prolonging the survival of patients with MM, as shown in our study. In addition, achieving undetectable MRD, especially in MM with high-risk CA, can overcome the dismal prognosis [13]. In this trial, the MRD-negative response rate was not significantly different in the multivariate analysis because many patients were not measured in the non-ASCT group. In our further study, we will measure the MRD when CR is achieved, even in patients who are ineligible for ASCT.

In this study, we found that ASCT can still be safely performed in MM patients over 65 years, as previously reported [4–9]. The CR rate in the ASCT group in this study was 30% after transplantation but increased to 60% with consolidation therapy. Additionally, in this study the induction therapy was shorter in the ASCT group and the time to transplant also tended to be shorter, which raised the concern that the stem cell collection rate would drop. Moreover, the pretreatment was reduced to conditioning regimen owing to concerns about TRM. Even with a reduced conditioning regimen at age 65 years or older, ASCT plus novel drug consolidation therapy was highly effective and similar to the IFM 2009 trial performed in younger patients (CR rates in IFM 2009 ASCT group: 59%, our study: 60%) [1]. Although it may be important to achieve a deep response after transplantation, whether this will lead to prolonged survival in the future remains controversial. The reason why some patients in the ASCT group did not achieve CR was that patients with less than very good partial response (VGPR) after ASCT were given maintenance therapy with lenalidomide or ixazomib without consolidation therapy and worsened without achieving CR. This finding indicates the importance of treatment aiming for a deep CR with MRD negativity in patients with high VGPR levels after ASCT, with combination therapy that employs novel agents, including anti-CD38 mAbs, and/or carfilzomib for consolidation therapy after ASCT.

The CR rate in the non-ASCT group was comparable to that in the MAIA group (DRd: CR rate 51%, 58%) [9]. In the non-ASCT group with patients 65 years and older, CR rates and PFS were similar to or better than the results of ASCT plus VRd therapy in younger patients in the IFM 2009 trial (IFM 2009 ASCT: mPFS, 50 months; non-ASCT group in this study: mPFS, 94.2 months) [1]. In the MAIA trial, 55% of the patients were 65–74 years old, but 43% were 75 years and older, with similar CR rates to what was obtained in the ASCT group in this study; therefore, DRd therapy may have a similar outcome as transplantation for fit patients even if they are over 75 years old [10].

Berrotti et al. showed that PFS was superior in the ASCT group than in the not-ASCT group for MM aged 65–74 years which is roughly the same age range as in our study; however, first-line treatment in the most patients of not-ASCT group in this study was bortezomib, melphalan, prednisone (VMP), with only 2/46 patients receiving the daratumumab base regimen [14]. This study is very different from our study, which included many anti-CD38 mAbs that are expected to respond better than bortezomib-based regimens.

Based on the results of this study, ASCT is becoming less significant in patients older than 65 years, but alkylating agents with ASCT are important treatments for patients with extramedullary disease (EMD) or plasma cell leukemia (PCL), and ASCT should be performed whenever possible in such cases [15–17]. It is necessary to consider which cases, other than EMD-MM and PCL, should be aggressively treated with ASCT.

This retrospective cohort study had some limitations. First, our study used a retrospective design and may therefore be susceptible to disadvantages such as patient selection bias. Furthermore, the treatment selection was left to the physician, and there was no set protocol for selection. Nevertheless, our study is significant because the not-ASCT group may have had worse patient background conditions than the ASCT group. Second, the relatively very small number of patients included in each molecular risk subgroup may have led to failure in distinguishing the prognostic differences between the two groups. Further investigation in prospective studies will be needed. Third, post-transplant treatment in the ASCT group was not standardized, which may have affected post-transplant prognosis.

In conclusion, ASCT is becoming less significant in patients older than 65 years because novel agents can achieve a good response without ASCT. Achievement of CR with or without ASCT appears to be most important for prolonging the survival of patients with MM. Even in ASCT-ineligible cases, combinations of novel agents such as daratumumab can provide results comparable to those of ASCT. Still, EMD-MM and PCL cases should be aggressively treated with ASCT.

## Data Availability

The datasets generated during and/or analyzed during the current study are available from the corresponding author on reasonable request.
